# The evolution of extravascular cardioverter defibrillator implantation: feasibility without general anaesthesia

**DOI:** 10.1093/europace/euag217

**Published:** 2026-08-19

**Authors:** Mauro Biffi, Hans Römers, Andrea Quaranta, Antonio Nicolò Izzo, Carmine Verde, Alberto Spadotto, Jippe Balt, Andrea Angeletti, Cristian Martignani, Igor Diemberger, Matteo Ziacchi, Lucas V A Boersma

**Affiliations:** IRCCS Azienda Ospedaliero-Universitaria di Bologna, Via Massarenti 9, 40138 Bologna, Italy; Cardiology Department, St. Antonius Hospital Nieuwegein, Koekoekslaan 1, 3435 CM Nieuwegein, The Netherlands; Cardiology Department, Amsterdam University Medical Center, Meibergdreef 9, 1105 AZ Amsterdam, The Netherlands; Dipartimento di Scienze Mediche e Chirurgiche, Istituto di Cardiologia, Università di Bologna, via Massarenti 9, 40138 Bologna, Italy; Dipartimento di Scienze Mediche e Chirurgiche, Istituto di Cardiologia, Università di Bologna, via Massarenti 9, 40138 Bologna, Italy; Dipartimento di Scienze Mediche e Chirurgiche, Istituto di Cardiologia, Università di Bologna, via Massarenti 9, 40138 Bologna, Italy; Dipartimento di Scienze Mediche e Chirurgiche, Istituto di Cardiologia, Università di Bologna, via Massarenti 9, 40138 Bologna, Italy; Cardiology Department, St. Antonius Hospital Nieuwegein, Koekoekslaan 1, 3435 CM Nieuwegein, The Netherlands; IRCCS Azienda Ospedaliero-Universitaria di Bologna, Via Massarenti 9, 40138 Bologna, Italy; IRCCS Azienda Ospedaliero-Universitaria di Bologna, Via Massarenti 9, 40138 Bologna, Italy; Dipartimento di Scienze Mediche e Chirurgiche, Istituto di Cardiologia, Università di Bologna, via Massarenti 9, 40138 Bologna, Italy; IRCCS Azienda Ospedaliero-Universitaria di Bologna, Via Massarenti 9, 40138 Bologna, Italy; Cardiology Department, St. Antonius Hospital Nieuwegein, Koekoekslaan 1, 3435 CM Nieuwegein, The Netherlands; Cardiology Department, Amsterdam University Medical Center, Meibergdreef 9, 1105 AZ Amsterdam, The Netherlands

## Abstract

**Aims:**

Extravascular implantable cardioverter-defibrillator (EV-ICD) is a novel non-transvenous system delivering defibrillation shocks and anti-tachycardia pacing through a substernal lead. Despite proven efficacy, implantation has traditionally required general anaesthesia and cardiothoracic surgical backup, limiting widespread adoption.

**Objectives:**

To evaluate feasibility, safety, and workflow efficiency of EV-ICD implantation under cardiologist-directed sedation without anaesthesiology support.

**Methods and results:**

Prospective observational study including 115 consecutive patients undergoing EV-ICD implantation across two European centres. Three procedural settings were explored sequentially: general anaesthesia (GA, *n* = 46), analgosedation without anaesthesiology supervision (AS, *n* = 28) and regional block with limited sedation (RBS, *n* = 41). The primary endpoint was successful implantation without conversion to respiratory or circulatory support. Secondary endpoints included procedure duration, procedure-related complications, and device performance at follow-up. Overall implantation success was 97% (112/115) in a predominantly young population (50 ± 17 years; 70% male). All procedures performed without anaesthesiology supervision were completed without conversion to GA or emergency support, with comparable complication rates across groups. RBS was associated with a 27-minute reduction in procedural duration vs. GA (*P* < 0.001) and earlier mobilization (97% ambulating within 3 h vs. 65% after 6 h with GA; *P* < 0.001). Over a median follow-up of 20 [10–30] months, appropriate ICD therapy was delivered in 13% of patients with 100% arrhythmia termination. Inappropriate therapy occurred in 8.9%, predominantly during the early learning phase.

**Conclusion:**

EV-ICD implantation can be safely and effectively performed under cardiologist-directed sedation without anaesthesiology supervision, achieving a fully electrophysiology-driven workflow. Procedural simplification substantially reduces resource utilization and may facilitate broader adoption of this technology.

## Graphical Abstract

**Graphical Abstract:**
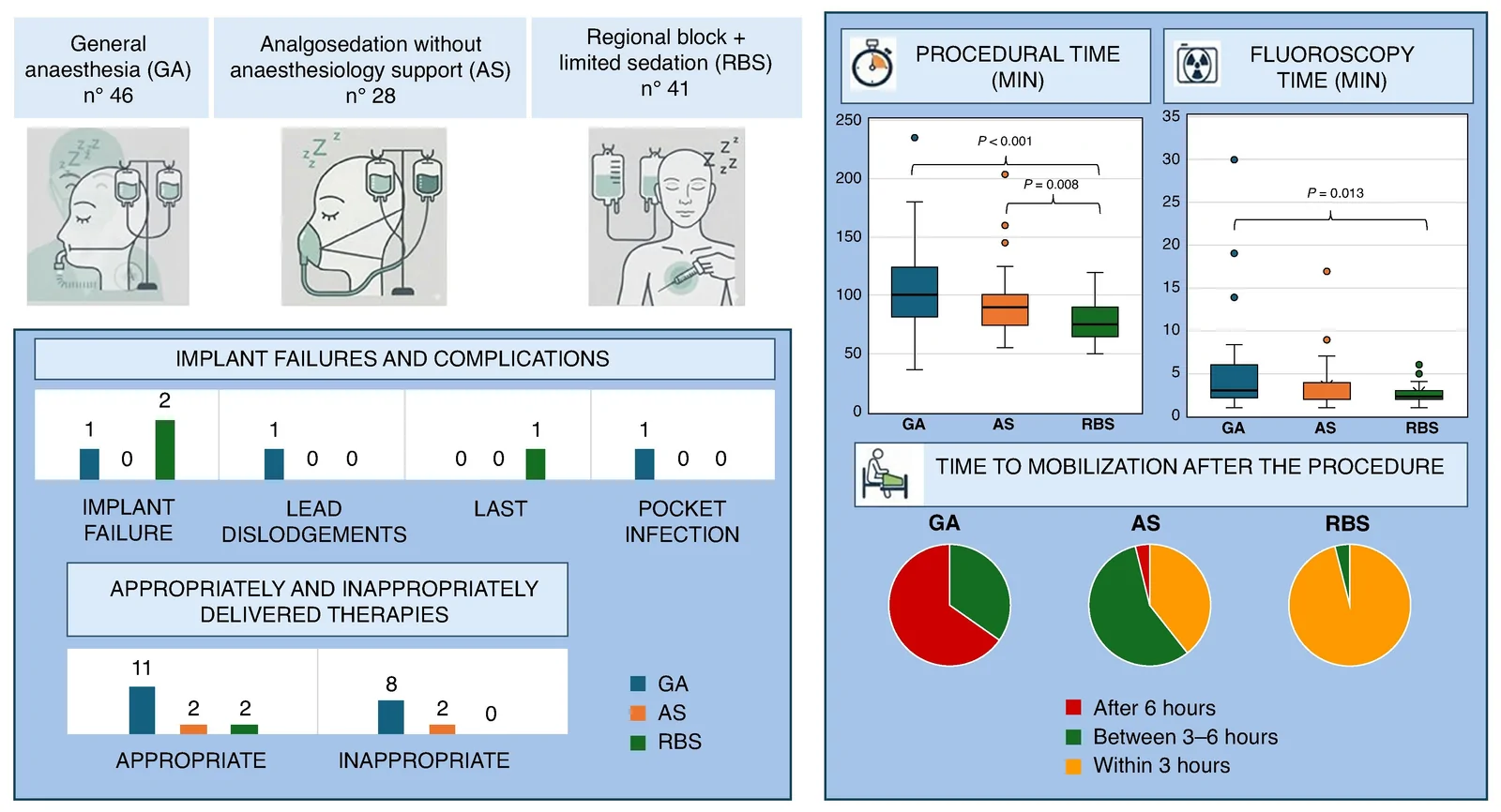

What’s New

- EV ICD implantation can be safely performed by cardiologists without anaesthesiology support, similarly to other advanced electrophysiological procedures (e.g. S-ICD implantation and AF ablation).
- A strategy based on regional block with on-demand sedation reduces overall sedative burden, enabling shorter procedures and faster post-procedural recovery compared with general anaesthesia.
- Simplification of the procedural setting may facilitate broader adoption of EV ICD implantation beyond high-volume centres.

## Introduction

The extravascular implantable cardioverter defibrillator (EV-ICD) is a novel non-transvenous cardioverter/defibrillator capable of defibrillation and anti-tachycardia pacing (ATP) owing to the intrathoracic lead placement in the anterior mediastinum.^1,2^ Despite proven efficacy and safety, its broad adoption remains limited by the implantation approach with oral intubation under general anaesthesia and by the recommendation of cardiothoracic surgery availability during the learning phase and later as a backup in the implantation centre.^1,3^ Early during the PIVOTAL trial phase, we prospectively planned at the University Hospital of Bologna a strategy to move from this resource-demanding setting towards a fully cardiologic one, firstly shifting from oral intubation to laryngeal mask respiratory support and then to deep sedation. Following the clinical availability of the Aurora ICD [Medtronic, Mounds View, MN] in 3rd quarter 2023, the experience was extended in collaboration with Amsterdam University at St. Antonius Hospital, Nieuwegein.

The aim of our experience was to prove that EV ICD can be accomplished with the same safety and efficacy under operator-directed nurse-administered sedation without anaesthesiology supervision like many other cardiologic procedures such as AF ablation or S-ICD implantation.^4–11^

## Methods

This is a prospective observational study reporting the outcome of EV ICD implantation in different settings, aiming to optimize the workflow and efficiency without compromising safety or efficacy. During the 5 years elapsed, we progressively modified the procedure setting, moving from a surgical intervention setting to an interventional electrophysiologic procedure not requiring anaesthesiology or respiratory support. The three operative settings which we explored are general anaesthesia (GA) with oral intubation (OIT) or assisted via laryngeal mask (LM), analgosedation without anaesthesiology support (AS) or regional block + limited sedation (RBS). The implantation could take place in a surgical OR, a hybrid room (HyR) or an electrophysiology room (EPR), depending on radiologic equipment quality and staff/room availability. Once GA was abandoned, scheduling was no longer dependent on anaesthesiology and room availability.

GA was conducted in 46 patients by an anaesthesiologist with full patient monitoring. AS and RBS without anaesthesiology support required an EKG, pulse oximeter, non-invasive BP, external defibrillator with thoracic patches, and patient monitoring by the Modified Observer’s Assessment of Alertness/Sedation (MOAA/S) scale.^7^ AS was achieved using intravenous fentanyl (40–100 mcg) administered at the time of sterile dressing, combined with local anaesthesia (lidocaine 200 mg), and continuous propofol infusion (dexmedetomidine in children below age 15). Sedation was titrated to achieve a level 1 on the Modified Observer’s Assessment of Alertness/Sedation (MOAA/S) scale^7,10^; level 0 was achieved in the event the patient could not tolerate blunt finger dissection. RBS consisted of a regional block at the upper rectus abdominis sheath and at the serratus plane, paired with limited sedation prior to the tunnelling steps. The regional block was achieved 20 min before entering the hybrid or the EP room by sterile injection of 100 mg diluted ropivacaine both at the upper rectus-transversus abdominis fascia and at the serratus plane (in the event of intermuscular ICD placement) under echographic guidance; superficial anaesthesia was achieved by lidocaine 200 mg before sterile dressing.^11–13^ Sedation was added after skin incision to allow comfortable lead tunnelling without respiratory depression at a MOAA/S level 1/0, according to different schemes:

- Fentanyl (50 up to 200 mcg) + etomidate (4 up to 40 mg) bolus (13 patients);
- Midazolam (2 mg) + ketamine (40 mg) bolus, ketamine repeated upon patients’ need up to 1.5 mg/kg (28 patients).

RBS has the advantage of minimizing sedation need and of enhancing pain management after the procedure, thus enabling fast patient mobilization with lesser use of painkilling medications.^12,13^ The choice of these drugs aims to minimize respiratory depression and workload, owing to the avoidance of histamine release (etomidate) and to airway smooth cell relaxation (ketamine). In nurse-administered under operator-directed sedation, drug dosage and delivery timing are decided by the physician, and the patient’s reassessment along the procedure guides the predefined administration protocol reported above. The operator doing the implant needs to be proficient with the sedation management, having been trained by anaesthesiologists in the use of drugs, and must have competence in the management of upper airways in emergency. Nurses are BLS-certified, experienced in non-invasive upper airways management, and trained to monitor the MOAA/S level of consciousness, the vital parameters, and breathing patterns. They are instructed to place the Guedel cannula in the event respiratory effort/signs of obstruction occur (snoring or S0_2_ drop below 90%) and to supplement oxygen via face mask or nasal cannula at low flow, modulating its amount and the oxygen fraction if needed. They can operate secretions aspiration in the event they develop. Monitoring patients’ consciousness status enables timely drug administration based on the procedure steps. Having been trained formerly with ICD defibrillation testing, electrical cardioversion of AF, S-ICD surgery, and AF ablation procedures, nurses were already experienced with drug administration and patient monitoring, and having already experienced 20 GA-assisted implants, they were familiar with the procedure steps and the sedation depth needed.

EV-ICD implantation techniques with the EV ICD have evolved in the past 6 years and have been previously described.^1–3,14,15^ In brief, a chest CT or MR is recommended before implantation to guide lead positioning in terms of lead location in front of the RV and length of tunnelling. Landmarks for lead tunnelling are generally identified as around 1 cm above the cardiac apex (proximal electrode, R2) and at the upper RVOT in the left-lateral view (distal electrode, R1) by placing a demo lead onto patients’ chest.^15^ To access the substernal space, an incision is made in-between the left costal margin and the xiphoid process. After blunt finger dissection, a sheath mounted on a dedicated tunnelling tool is advanced beyond the rectus fascia and ensured to be in proximity/direct contact with the posterior surface of the sternum via fluoroscopy, possibly in front of the RV.^15^ Once the path is created, the EV-ICD lead is placed in the substernal space: its positioning is evaluated by measuring the ventricular signal parameters (R-wave ≥1 mV in R1-R2 vector and P wave non-detectable or at least ≤0.2 mV). In case of inadequate signal, the lead is repositioned to achieve appropriate sensing parameters. The proximal end of the lead is then tunnelled to a left lateral subcutaneous or intramuscular pocket (according to operators’ preference) near the mid axillary line. Defibrillation testing (DFT) is performed unless clinical conditions recommend otherwise. After implantation, the patients are mobilized and allowed to walk as soon as permitted by clinical conditions. Device interrogation and 2-view standing chest x-rays are taken before hospital discharge.

Informed consent and ethical committee approval were obtained at each individual institution level as single-centre practice and regulations. The current study was performed in observance of the Declaration of Helsinki.

### Data definition and collection

De-identified baseline clinical data, pre- and peri-procedural data, and follow-up data were collected by the participating centres in accordance with a predefined database.

Data from the last imaging tests available prior to the index procedure [EKG, coronary angiography, *trans*-thoracic echo (TTE) and/or magnetic resonance imaging (MRI)] were available as per clinical practice. The EV-ICD lead final position was collected as well, defined with respect to the sternum as substernal (beneath the sternum), parasternal (placed along the left sternal border), or laterosternal (placed 5–15 mm to the left of the sternal border).^15^

#### Measurements

All procedure-associated complications were recorded, as well as device-related events until the last available follow-up. Electrical measurements at implantation (R wave amplitude, P wave detectability and amplitude, pacing and shock impedance, defibrillation strength and polarity) were collected. Procedural time was defined as the time elapsed since the 1st incision until the last suture. Device performance at follow-up in terms of electrical parameters and appropriate and inappropriate therapy delivery was also recorded.

Major adverse events were defined as death, complications leading to new interventions or transient/permanent disability, permanent loss of device function due to device dysfunction, device-associated infection requiring hospitalization and/or system revision, and bleeding requiring transfusion or operative management. Appropriate therapies were defined as the delivery of anti-tachycardia pacing, shocks, or a combination of the two in response to a properly detected ventricular arrhythmia (VA). Inappropriate therapy delivery (IAT) was defined as the delivery of ICD therapies as a result of a supraventricular tachycardia, oversensing of either cardiac or non-cardiac signals, or any other reason leading to a therapy in the absence of a clinical VA.

The study endpoint was the capability to complete the EV ICD implantation without conversion to respiratory or circulatory support because of unwanted effects of the sedation strategy/GA. Secondary endpoints were procedure time, number and type of procedure-related complications and occurrence of appropriate and inappropriate ICD interventions at follow-up.

### Statistical analysis

Continuous variables are presented as mean ± standard deviation (SD) or median (interquartile range [IQR]), and categorical variables as counts (percentages). Categorical variables were compared across the three groups using the χ^2^ test; Fisher’s exact test was applied when appropriate. In the presence of a statistically significant overall test, post-hoc pairwise comparisons were performed with Bonferroni correction. Continuous variables were compared across the three groups using the Kruskal–Wallis test, which was selected given the limited sample size within groups and the resulting uncertainty regarding the validity of parametric assumptions, irrespective of the observed distribution. Significant overall differences were further explored using pairwise Wilcoxon rank-sum tests with Bonferroni adjustment. All analyses were performed using Stata/BE version 19.0 (StataCorp, College Station, TX, USA).

## Results

A total of 115 patients underwent an attempt at EV-ICD implantation, with 112 (97.4%) ultimately receiving the device (*Table 1* and *Table 2*): two had a < 0.9 mV R wave despite several lead tunnelling attempts (one GA and one RBS, both with ischaemic heart disease), and one (RBS, ischaemic heart disease) aborted the procedure because of pneumothorax, which recovered uneventfully, but made it impossible to complete lead placement (lead unstable with unreliable measurements). These three patients received a transvenous ICD. As reported in *Table 1*, the population was young (50 ± 17 years) with 69.6% of males and a predominance of non-ischaemic cardiomyopathies, arrhythmogenic being the leading one (see Supplementary material online, *Table S1*); primary prevention accounted for 78.4% of patients. Two patients were referred because of repeated IATs delivered by an S-ICD which could not be fixed owing to poor signal in all vectors at follow-up (one Brugada, one dilated cardiomyopathy), while 12 more patients failed the screening test for the S-ICD (one Brugada, two hypertrophic, two left-dominant and six biventricular arrhythmogenic cardiomyopathy, one dilated cardiomyopathy). Seven implantations took place in a surgical OR (6.1%), while 78 (67.8%) in a HyR, and 30 (26.1%) in an EPR; 46 (40%) occurred under GA, 28 (24.3%) in AS and 41 (35.7%) in RBS. All procedures were done by four different cardiologists. Cardiac surgery backup ceased after the 10th procedure; a cardiac surgeon is available for consultation in the room within 5 min in both our institutions. None of the procedures made without anesthesiologist support required respiratory/circulatory support or conversion to GA: all were completed in a fully cardiologist-driven setting. As reported in *Table 2*, there were no differences in procedure-related adverse events across the three operative settings: an uneventful pneumothorax and a lidocaine-related toxicity (following local analgesia) requiring 10 mg i.v. diazepam occurred in an RBS patient. A single infection occurred in a GA patient and dictated system removal at follow-up. A rather common unwanted event was pneumomediastinum in 6 patients (*Table 2*), which was detected by fluoroscopy as an air layer between the sternum and the pericardium in a left-lateral radiologic view. In four cases was perceived by the operator due to air sucking in the mediastinum caused by negative intrathoracic pressure during transient deep sedation at MOAA/S level 0 (one GA on laryngeal mask, two AS and one RBS patients). Though impacting on the electrical parameter measurements (smaller R wave and higher pacing/shock impedance than expected), this was not a clinical problem: the procedure completed uneventfully and pneumomediastinum resolved within 24 h without causing symptoms or prolongation of hospital stay. The behaviour of electrical parameters at implantation in the event of pneumomediastinum is shown in *Figure 1*. ICD placement was intermuscular in 78/112 patients (69.6%) and subcutaneous in 34 (30.3%).

**Figure 1:**
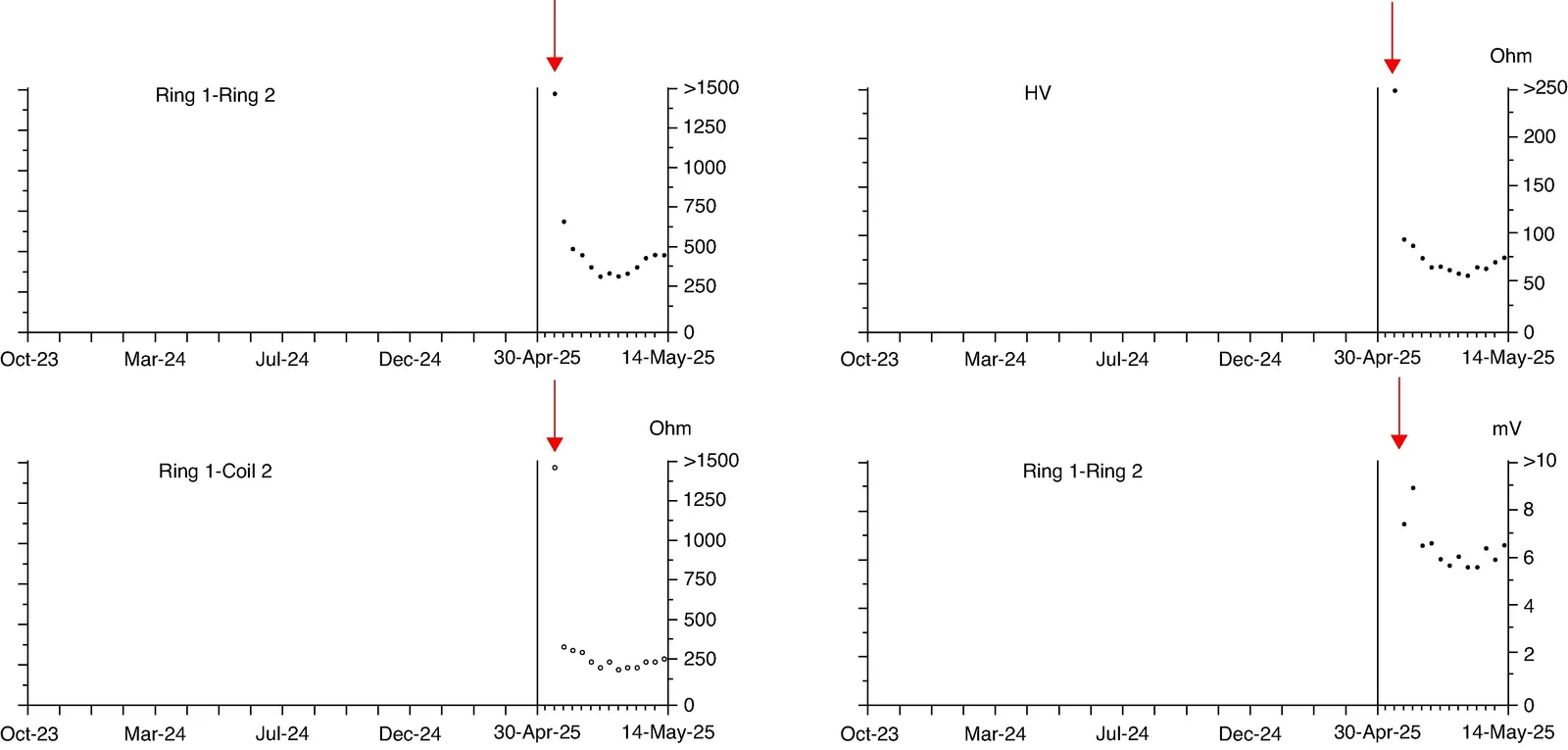
Example of pneumomediastinum: red arrows indicate High Voltage (HV), sensing/pacing vector (Ring1—Ring2), and ring 1—coil 2 vector impedance, sensing amplitude as measured at implantation and in the following 13 days. Note that impedance is out of range, and returns to normal range within 24 h.

**Table 1:** Patient characteristics

Patient characteristics	All (115)	GA (*N* = 46)	AS (*N* = 28)	RBS (*N* = 41)	*P*
Age—years (SD)	49.9 ± 17.3	54.4 ± 16.2	40.4 ± 18.0	51.4 ± 15.8	0.002^a^
Male—no. (%)	80 (69.6)	31 (67.4)	21 (75.0)	28 (68.3)	ns
BMI—kg/mq	24.9 ± 4.4	25.2 ± 4.8	24.1 ± 3.7	25.0 ± 4.2	ns
*Comorbidities*					
Diabetes—no. (%)	10 (8.7)	5 (10.9)	1 (3.6)	4 (9.8)	ns
Hypertension—no. (%)	30 (26.1)	12 (26.1)	4 (14.3)	14 (34.1)	ns
Renal insufficiency—no. (%)	5 (4.3)	3 (6.5)	1 (3.6)	1 (2.4)	ns
Ejection fraction—%	44 ± 15	39 ± 15	51 ± 12	43 ± 16	0.005^b^
Atrial fibrillation—no. (%)	18 (15.6)	8 (17.4)	3 (10.7)	7 (17.1)	ns
Antiplatelets—no. (%)	24 (20.9)	12 (26.1)	3 (10.7)	9 (22.0)	ns
Anticoagulants—no. (%)	29 (25.2)	12 (26.1)	5 (17.9)	12 (29.3)	ns
*Indication for ICD* primary prevention—no. (%)	91/115 (79.1)	36 (78.3)	23 (82.1)	32 (78.0)	ns
Chest CT or MRI Available—no. (%)	94 (81.7)	33 (71.7)	28 (100)	33 (80.5)	0.003**^b^**
Non-ischaemic cardiomyopathy—no. (%)	86 (74.8)	31 (67.4)	26 (92.9)	29 (70.7)	0.038**^b^**
Procedural characteristics					
*Intervention room* • EP lab—no. (%) • Surgical OR—no. (%) • Hybrid OR—no. (%)	30 (26.1)	14 (30.4)	0	16 (39.0)	<0.001**^c^**
	7 (6.1)	7 (15.2)	0	0	
	78 (67.8)	25 (54.3)	28 (100)	25 (61.0)	

^a^The overall difference is primarily driven by the younger age observed in group AS, whereas no significant difference was observed between GA and RBS in Bonferroni-adjusted pairwise comparisons.

^b^The overall difference was primarily driven by the comparison between GA and AS, while group RBS showed intermediate values without statistically significant differences from the other groups.

^c^An exploratory multinomial logistic regression model was attempted; however, reliable estimates could not be obtained due to complete separation, as all procedures in the AS group were exclusively performed in a hybrid operating room.

AS, analgosedation; BMI, body mass index; EP, electrophysiology; GA, general anaesthesia; ICD, implantable cardioverter defibrillator; OR, operating room; RBS, regional block + sedation; SD, standard deviation

**Table 2:** Procedure outcome

	All (*N* = 115)	GA (*N* = 46)	AS (*N* = 28)	RBS (*N* = 41)	*P*
Procedure duration—min (SD)	93 ± 31	105 ± 35	95 ± 32	78 ± 18	<0.001^a^
Fluoroscopy time—min (SD)	3.8 ± 3.8	4.9 ± 5.3	3.7 ± 3.4	2.8 ± 1.3	0.049^b^
Patients’ mobilization • Within 3 hours (%) • Between 3 and 6 hours (%) • After 6 hours (%)	52 (45.2)	0	12 (42.8)	40 (97.6)	<0.001^c^
	32 (27.8)	16 (34.8)	15 (53.6)	1 (2.4)	
	31 (27.0)	30 (65.2)	1 (3.6)	0	
Implant failure (%)	3 (2.6)	1 (2.1)	0	2 (4.9)	ns
Inadequate parameters	2 (1.7)	1 (2.1)	0	1 (2.4)	ns
Unreliable lead position	1 (0.8)	0	0	1 (2.4)	ns
Complications	3 (2.6)	2 (4.3)	0	1 (2.4)	ns
Lead dislodgement	1 (0.9)	1 (2.1)	0	0	ns
LAST	1 (0.9)	0	0	1 (2.4)	ns
Pocket infection	1 (0.9)	1 (2.1)	0	0	ns
Pneumothorax with unreliable lead position	1 (0.9)	0	0	1 (2.4)	ns
Unwanted events					
Pneumomediastinum	6 (5.2)	1 (2.1)	2 (7.1)	2 (4.9)	ns

^a^The overall difference was driven by significantly shorter procedural times in RBS, whereas GA and AS showed comparable procedural durations.

^b^Pairwise comparisons with Bonferroni correction revealed a significantly shorter fluoroscopy time in RBS compared with GA, while no significant differences were observed between the other groups.

^c^All pairwise between-group comparisons were statistically significant after Bonferroni correction.

AS, analgosedation; FU, follow-up; LAST, local analgesia systemic toxicity; GA, general anaesthesia; RBS, regional block + sedation.

According to the operative settings, there was a clinically significant reduction in the use of resources moving from GA to RBS, as no anaesthesiologist was required, and an average 27 min reduction in procedure duration occurred. In addition, a small but statistically significant reduction in fluoroscopy time (approximately 2 min) was observed (*Table 2*, *Figure 2*). When comparing the first 10 vs. the last 10 cases done under GA at each centre, no procedure duration difference was observed (see Supplementary material online, *Table S2*), while a significant reduction in fluoroscopy time occurred when considering the first against the last five cases. No learning-curve effect was noted for patients operated under RBS (see Supplementary material online, *Table S2*).

**Figure 2:**
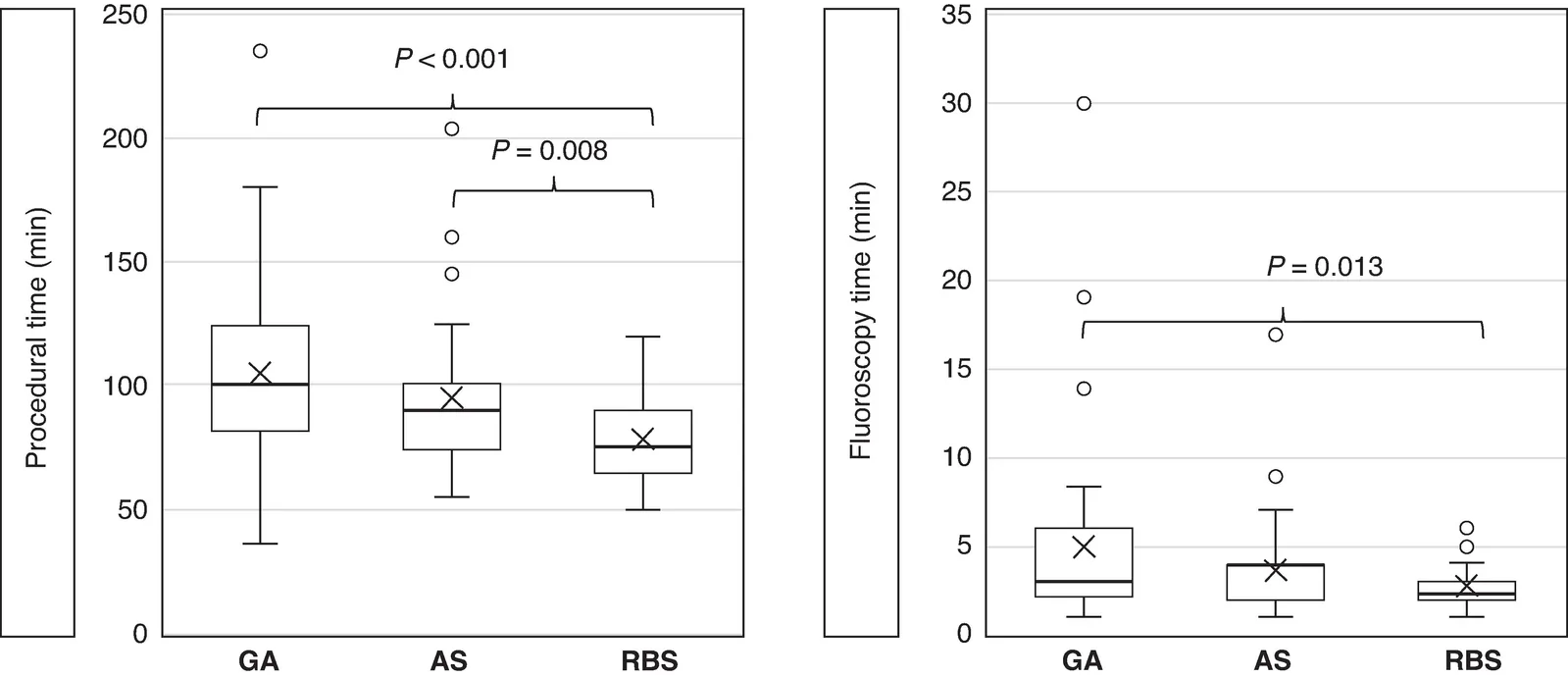
Distribution of procedural time and fluoroscopy exposure across the three implantation settings. AS, analgosedation; GA, general anaesthesia; RBS, regional block + sedation.

40/41 (97.6%) patients operated in the RBS setting stood up and walked within 2 and 3 h from the implantation, whereas 15/28 (53.6%) of the AS operated walked between 3 and 6 h, and 30/46 (65.2%) % of GA patients walked after 6 h up to the next morning (*Table 2*).

Median follow-up was significantly different between the earliest cohort implanted under GA and the most recent implanted under RBS, which can explain the larger incidence of appropriate therapy delivery (*Table 3*). The inappropriate therapy delivery was limited to the early phase of both centres (*Table 3*), while it was not observed since 2024 onward.

**Table 3:** Appropriate and inappropriate therapies across the three implantation settings

	All (*N* = 112^a^)	GA (*N* = 45^a^)	AS (*N* = 28)	RBS (*N* = 39^a^)	*P*
Median follow-up (months)	20 (IQR 10–30)	*54* *(IQR 27–59)*	*20* *(IQR 13–25)*	*8* *(IQR 3–10)*	<0.001^b^
Appropriately delivered therapy	15 (13.4)	11 (24.4)	2 (7.1)	2 (5.1)	0.025^c^
• ATP	4 (3.6)	*2* (*4.4)*	*0*	*2* (5.1)	*ns*
• Shock	4 (3.6)	*4* (*8.9)*	*0*	*0*	*ns*
• ATP + shock	7 (6.2)	*5* (*11.1)*	*2* (7.1)	*0*	*ns*
Shock efficacy	100%	*100%*	*100%*		
Inappropriate delivered therapy	10 (8.9)	8 (17.8)	2 (7.1)	0	0.028^d^
• P wave oversensing	1 (0.9)	*1* (*2.2)*	*0*	*0*	*ns*
• Myopotential oversensing	5 (4.5)	*4* (*8.8)*	*1* *(3.6)*	*0*	*ns*
• T-wave oversensing	1 (0.9)	*1* (*2.2)*	*0*	*0*	*ns*
• SVT	3 (2.7)	*2* (*4.4)*	*1* *(3.6)*	*0*	*ns*

^a^The analysis was restricted to patients who underwent device implantation.

^b^All pairwise between-group comparisons were statistically significant after Bonferroni correction

^c^The overall difference was driven by a higher rate of appropriate treatment in GA compared with RBS, whereas group AS showed intermediate values without statistically significant differences

^d^The rate of inappropriate treatment differed across groups; however, pairwise comparisons did not show significant differences after Bonferroni correction.

AS, analgosedation; ATP, anti-tachycardia pacing; GA, general anaesthesia; IQR, interquartile range; RBS, regional block + sedation; SVT supraventricular tachycardia

The 112 patients with a successful placement of an EV-ICD were followed for an average of 20 [10–30] months, with 97/112 (86.6%) having at least 6 months follow-up as of February 2026. During this follow-up time, 15 (13.4%) patients experienced appropriate therapy delivery (*n* = 4 ATP only; *n* = 4 shock only; *n* = 7 ATP and shock), as reported in *Table 3*.

During follow-up, inappropriate therapy was delivered in 10/112 (8.9%) patients, with a higher incidence in the GA group compared with AS and RBS (17.8% vs. 7.1% vs. 0%, respectively). Although this difference is statistically significant (*P* = 0.028), it must be interpreted cautiously, particularly in light of the shorter RBS follow-up, the experience acquired in ICD programming and the availability of the P-wave oversensing algorithm since the PIVOTAL trial onwards. Myopotential oversensing was the leading cause of inappropriately delivered therapies, accounting for 5 patients (4.5%, 3/78 in intermuscular and 2/34 in subcutaneous device placements), followed by supraventricular tachycardia in 3/112 patients (2.7%). Less frequent mechanisms included P-wave oversensing and T-wave oversensing, each observed in 1/112 patients (0.9%) as reported in *Table 3*. Reprogramming the sensing vector from Ring-can to Ring1-Ring2 and increasing the oversensing prevention level from nominal (3) to 5 solved myopotential oversensing, while reprogramming SVT discriminators and the sensing threshold decay delay fixed SVT incorrect detection and T-wave oversensing. No patient had further inappropriate therapy delivery after reprogramming occurred following the first event.

## Discussion

The present study describes the progressive evolution of extravascular ICD implantation from a resource-intensive surgical procedure to a fully electrophysiology-driven intervention, demonstrating that EV-ICD implantation can be safely and effectively performed without GA or anaesthesiology supervision. Our experience shows that, through a stepwise optimization of sedation strategy, procedural workflow and operator expertise, EV-ICD implantation may ultimately resemble other advanced electrophysiological procedures routinely performed in the EP laboratory, such as AF ablation or S-ICD implantation.^4–11,16,17^

### Transition from surgical to electrophysiologic setting

The EV-ICD was initially introduced as a hybrid technology, combining the advantages of non-transvenous defibrillation with ATP capability, at the cost of a more complex implantation approach requiring general anaesthesia and surgical backup. While these precautions were justified during the early feasibility phases, they represent a relevant barrier to widespread adoption in daily clinical practice. Our data demonstrate that, once the learning curve is overcome, neither general anaesthesia nor routine surgical support is mandatory to achieve procedural success and safety. Likewise, Schiavone *et al*. recently reported equivalence of GA and deep sedation management in a large propensity score-matched population of S-ICD recipients.^11^ Scaglione *et al*. had indeed previously reported that S-ICD implantation does not require GA and can be accomplished by hypnotic communication and local analgesia with reduced pain perception, reduced anxiety, and lesser use of analgesic drugs compared to conventional AS.^16^ Furthermore, Scaglione *et al*. have recently extended hypnotic communication to pulsed-field ablation of AF, proving high patients’ tolerability and bailout shift to EP-managed sedation (ketamine 1.5–2 mg/kg or dexmedetomidine 1 mcg/kg/h) in only 30% of cases.^17^

Across the three operative settings explored—GA, AS and RBS—no differences were observed in acute procedural success or in the incidence of major adverse events (*Table 2*). Importantly, none of the procedures performed without anaesthesiology support required conversion to general anaesthesia or emergency respiratory or circulatory assistance, confirming that EV-ICD implantation is feasible under cardiologist-directed sedation when appropriate monitoring and emergency equipment are available. The administration of anaesthetic agents by cardiologists trained to avoid severe respiratory depression has proved safe as in previous experiences with AF ablation and S-ICD implantation.^4–11,16,17^ Rectus abdominis block combined with sedation on demand was the less time-consuming approach, with the best post-operative pain control and patients’ recovery time, enabling a 27 min-shorter procedure and faster patients’ mobilization (*Table 2*, *Figure 2*). Drug infusion titration and closer patient monitoring made AS less time-efficient and more demanding compared to RBS (*Table 2*, *Figure 2*).

### Sedation strategy and procedural safety

One of the key findings of this study is that both AS and RBS strategies provide adequate patient comfort while preserving procedural safety, with the caveat to avoid upper airway obstruction during phases of deep sedation at MOAA/S level 0, which can favour pneumomediastinum with transiently unreliable measurement of the electrical parameters (*Figure 1*). Compared to AS, RBS further reduced the need for deep sedation, improving post-procedural pain control and enabling earlier patients’ mobilization. Pneumomediastinum shall not be considered a procedure-related complication but rather an unwanted event occurring predominantly during the learning phase of each setting and in association with transient deep sedation. This finding highlights the importance of maintaining spontaneous respiration without excessive inspiratory effort during lead tunnelling when occurring under AS or RBS. Notably, all cases resolved spontaneously within 24 h with full recovery of electrical parameters (*Figure 1*) and did not translate into clinical complications or device malfunction, supporting the benign nature of this phenomenon. In COPD/obese/OSAS patients, lead tunnelling should take place after minimizing upper airway obstruction, for instance by placing a Guedel cannula during MOAA/S level 0 if snoring or increased respiratory effort is detected. Minimization of respiratory effort is enhanced by the use of ketamine or etomidate, which do not increase respiratory work while providing adequate sedation.

### Workflow efficiency and resource utilization

The progressive shift from GA to RBS was associated with a clinically meaningful reduction in procedural duration and resource utilization, as observed for PVI procedures in the large EU-PORIA registry.^9^ Avoiding anaesthesiology involvement allowed greater scheduling flexibility and reduced dependency on operating room or hybrid room availability. Moreover, earlier patient mobilization and reduced post-procedural pain translated into a more efficient post-implantation course, which may have relevant implications for hospital logistics and costs.

These aspects are particularly relevant for high-volume centres and may represent a key driver for broader EV-ICD adoption. At the same time, simplification of the procedural workflow may also enable the safe implementation of this technology in lower-volume hospitals, by reducing organizational complexity and resource requirements.

### Device performance and follow-up outcomes

Despite the heterogeneity in follow-up duration among implantation cohorts, device performance was consistent with previous EV-ICD reports.^1–3^ Appropriate therapies were effective in all cases, with 100% arrhythmia termination, confirming the reliability of both ATP and shock delivery in real-world conditions. Inappropriate therapies were similar to large studies^1,2^ and largely confined to the early learning phase, supporting the notion that optimization of sensing vectors and of detection/discriminators programming plays a critical role in minimizing oversensing-related events. Indeed, only 2/67 (3.7%) patients implanted with AS or RBS since October 2023 had inappropriate therapy delivery (*Table 3*), reflecting more likely the improvement in lead placement and in device programming rather than a role of the implantation strategy (GA vs. non-GA). Of note, no patient had relapsing inappropriate therapy delivery after the first one, thanks to device reprogramming.

Myopotential oversensing was not associated with intermuscular can placement when the Ring1-Ring2 sensing vector is programmed and required device reprogramming from Ring-to-Can to Ring1-Ring2 detection but not system revision. This observation may support the use of intermuscular pockets when feasible, particularly in thin-habit or younger and physically active patients.^18^

### EV-ICD in cardiomyopathies and inherited arrhythmia syndromes

A relevant proportion of our population consisted of young patients with arrhythmogenic or hypertrophic cardiomyopathy and inherited arrhythmogenic diseases (see Supplementary material online, *Table S1*), who often represent challenging candidates for conventional ICD systems or for S-ICD in terms of eligibility and may require ATP therapy.^18–25^ In these patients, the EV-ICD offers several advantages, including avoidance of transvenous leads and availability of ATP, features particularly appealing in younger patients with long life expectancy and elevated risk of lead-related complications.^18,19^

Moreover, EV-ICD proved to be a valuable alternative in patients who failed S-ICD screening or experienced inappropriate therapies due to poor sensing vectors, highlighting its complementary role within the current ICD armamentarium rather than a competing technology.^21–25^

### Learning curve and generalizability

All procedures were performed by a limited number of experienced electrophysiologists, and outcomes likely reflect the benefits of a structured learning curve. However, the consistency of results across two centres and different procedural environments suggests that the described workflow can be reproduced in other institutions with adequate training and case selection. The early discontinuation of routine surgical backup further supports the maturity of the implantation technique.^15,26^ Altogether, these findings may facilitate the future adoption of EV-ICD implantation beyond high-volume centres, including hospitals with lower procedural volumes.

### Study limitations

This study is observational in nature and involves a relatively limited sample size, with unequal follow-up duration among implantation cohorts. Long-term outcomes beyond the first year remain to be fully characterized. Additionally, the absence of a randomized comparison between sedation strategies limits definitive conclusions regarding superiority; nevertheless, the consistency of safety outcomes across groups strengthens the overall message.

### Conclusion and clinical implications

Our findings support a paradigm shift in EV-ICD implantation, moving from a surgically oriented procedure to a fully electrophysiological intervention, like in many other electrophysiological procedures^4–11,16,17^ . This evolution has the potential to significantly reduce procedural complexity, resource utilization, and barriers to adoption, while preserving safety and efficacy.

## Supplementary Material

euag217_Supplementary_Data

## Data Availability

The data underlying this article will be shared on reasonable request to the corresponding author.
