# Supplementary material for: The evolution of extravascular cardioverter defibrillator implantation: feasibility without general anaesthesia
Source: Europace. 2026 Aug 19;28(10):euag217. doi: 10.1093/europace/euag217 (PMC13669631; doi:10.1093/europace/euag217)
Supplement: Supplementary file 1 [file euag217_supplementary_data.docx]

**SUPPLEMENTARY MATERIAL**

Table S1. Underlying disease etiology

| Characteristic | All  (n=115) | GA  (n=46) | AS   (n=28) | RBS   (n=41) |
| --- | --- | --- | --- | --- |
| Ischemic heart disease | 29 (25.2%) | 15 (32.6%) | 2 (7.1%) | 12 (29.3%) |
|  |  |  |  |  |
| *Non-ischemic cardiomyopathy* | 86 (74.8%) | 31 (67.4%) | 26 (92.9%) | 29 (70.7%) |
| - ACM | 27 (23.5%) | 9 (19.6%) | 9 (32.1%) | 9 (22.0%) |
| *Right ventricular ACM* | 5 (4.3%) |  | 1 (3.5%) | 4 (9.8%) |
| *Left ventricular ACM* | *12* (10.4%) | 8 (17.4%) | 2 (7.1%) | 2 (4.9%) |
| *Biventricular ACM* | 10 (8.7%) | 1 (2.2%) | 6 (21.4%) | 3 (7.3%) |
| - Dilated | 18 (15.7%) | 10 (21.7%) | 1 (3.6%) | 7 (17.1%) |
| - Hypertrophic | 17 (14.8 %) | 4 (8.7%) | 8 (28.6%) | 5 (12.2%) |
| - Brugada Syndrome | 5 (4.3%) | 2 (4.3%) | 2 (7.1%) | 1 (2.4%) |
| - Idiopathic VF | 5 (4.3%) | 1 (2.6%) | 3 (10.7) | 1 (2.4%) |
| - Myocarditis | 2 (1.7%) | 1 (2.6%) | 0 | 1 (2.4%) |
| - Non-compaction dilated | 1 (0.9%) | 0 | 0 | 1 (2.4%) |
| - Duchenne cardiomyopathy | 2 (1.7%) | 0 | 2 (7.1%) | 0 |
| - Others | 9 (7.8%) | 4 (8.7%) | 1 (3.6%) | 4 (9.8%) |

ACM, arrhythmogenic cardiomyopathy; AS, analgosedation; GA, general anesthesia;  RBS, regional block + sedation.; VF, ventricular fibrillation

Table S2. Learning curve in EV ICD implantation.

|  | Patients | GA | P value | RBS | P value |
| --- | --- | --- | --- | --- | --- |
|  |  |  |  |  |  |
| Procedure Duration * |  |  |  |  |  |
| (minutes) | First 10 | 100.6±37 | NS | 75.9±18 | NS |
|  | Last 10 | 100.9±19 |  | 79.7±22 |  |
|  |  |  |  |  |  |
|  | First 5 | 103.4±40 | NS | 73.1±16 | NS |
|  | Last 5 | 97.6±15 |  | 87.3±24 |  |
|  |  |  |  |  |  |
| Fluoroscopy Time * |  |  |  |  |  |
| (minutes) | First 10 | 4.8±3.7 | NS | 2.7±1.4 | NS |
|  | Last 10 | 4.0±3.1 |  | 2.8±1.3 |  |
|  |  |  |  |  |  |
|  | First 5 | 7.7±4.1 | 0.021 | 2.6±1.3 | NS |
|  | Last 5 | 3.2±1.7 |  | 3.1±1.4 |  |
|  |  |  |  |  |  |
|  |  |  |  |  |  |

GA = general anaesthesia; RBS = rectus abdominis block + limited sedation.

* The last 10 patients done at Bologna Hospital in GA were assisted on laryngeal mask
